# Editorial: Revolutionizing obstetric and pediatric pharmacology through artificial intelligence and digital health innovations

**DOI:** 10.3389/fphar.2026.1955915

**Published:** 2026-10-28

**Authors:** Jeffrey S. Barrett, Catherine M. Sherwin, Samuel L. SewardJr

**Affiliations:** 1 Genmab US, Inc., Plainsboro, NJ, United States; 2 Internal Medicine, UWA Medical School, The University of Western Australia, Perth, WA, Australia; 3 Department of Experimental and Clinical Pharmacology, College of Pharmacy, University of Minnesota, Minneapolis, MN, United States; 4 Differentia Biotech Ltd, South San Francisco, CA, United States; 5 Department of Pharmacology and Toxicology, Wright State University Boonshoft School of Medicine, Dayton, OH, United States; 6 Department of Medicine, Icahn School of Medicine at Mount Sinai, New York, NY, United States

**Keywords:** artificial intelligence, clinical decision support, digital health, machine learning, obstetric pharmacology, pediatric pharmacology, pharmacokinetics, precision dosing

## Introduction

Obstetric and pediatric pharmacology remain among the most evidence-poor areas of clinical medicine. Pregnancy and childhood both drive substantial, poorly characterized changes in drug disposition (Pariente et al., 2016; Chapron et al., 2022), leaving clinicians reliant on empirical, often weight-based dosing that does not reflect true patient variability. Artificial intelligence, pharmacometrics, and digital health tools are increasingly proposed as ways to close this gap (Darwich et al., 2021). However, their value depends entirely on rigorous, population-specific evidence rather than on general enthusiasm for the technology. These tools span personalized dosing and pharmacometrics, predictive risk analytics, intelligent prescription screening, and digital monitoring and decision-support systems, and they offer a route to bridging historical data gaps in pregnant and pediatric populations.

Evidence of the progress in this field is highlighted in this Research Topic and in this editorial. Six original contributions are collected here: a population pharmacokinetic-machine learning hybrid model for linezolid dosing in critically ill children (https://doi.org/10.3389/fphar.2026.1817282); predicting serum phosphate levels in very preterm infants from routinely collected data (https://doi.org/10.3389/fped.2026.1881474); a consumer smartwatch for detecting spinal anesthesia-induced hypotension during cesarean delivery (https://doi.org/10.3389/fmed.2025.1686637); predicting intrapartum labor analgesia efficacy from preoperative indicators (https://doi.org/10.3389/fmed.2026.1794753); nalbuphine-ropivacaine infiltration and post-neuraxial puncture-site pain (https://doi.org/10.3389/fmed.2026.1908970); and the construction of China’s first Pediatric Rational Medication Intelligent Decision System (https://doi.org/10.3389/fped.2026.1736268). Read together, they trace a path from routinely collected clinical data to individualized prediction, real-time bedside monitoring, and system-level implementation (Figure 1). Collectively, these studies demonstrate that the greatest opportunity for AI in obstetric and pediatric pharmacology lies not in replacing clinical judgment, but in augmenting decision-making by integrating routinely collected clinical data with pharmacological knowledge.

**FIGURE 1:**
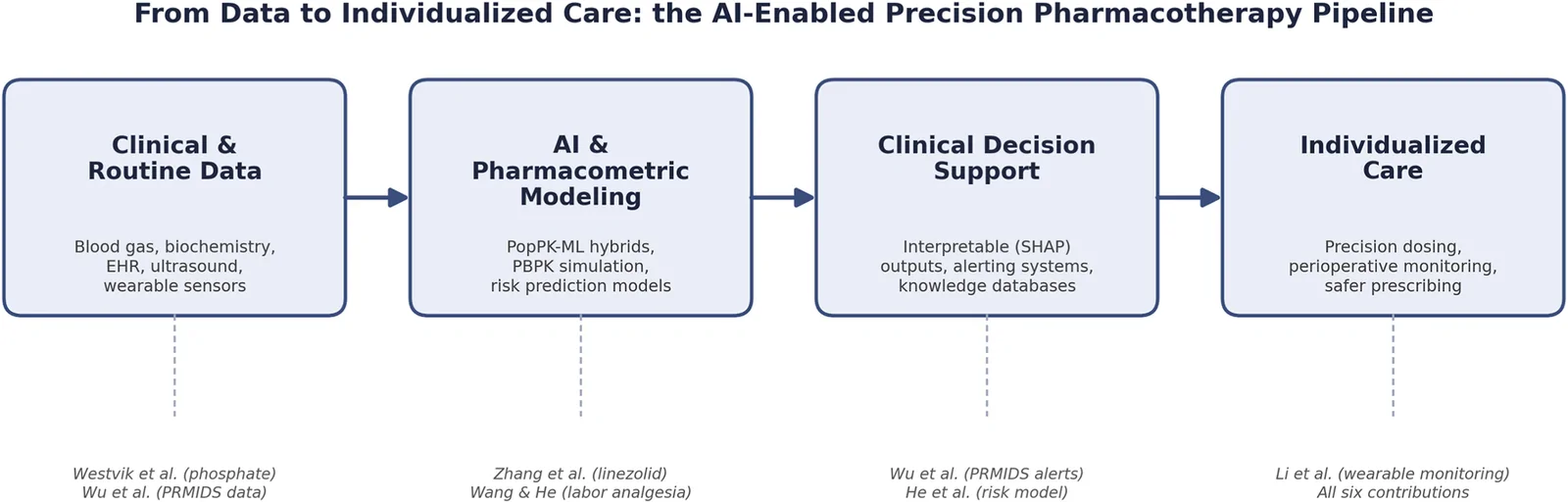
From Data to Individualized Care: conceptual pathway of the AI-enabled precision pharmacotherapy pipeline, from clinical and routine data through AI and pharmacometric modeling and clinical decision support to individualized obstetric and pediatric care, mapped to the six contributions in this Research Topic.

## Individualized prediction and dosing

Zhang et al. combined a population pharmacokinetic model of linezolid with machine learning in critically ill children, using SHapley Additive exPlanations (SHAP) to trace predictions back to clearance, dose, weight, and renal function. Notably, the authors flag their own strong validation metrics as optimistic, since the pharmacokinetic parameters used as model inputs were derived from the same concentration data being predicted, a transparency that usefully clarifies what leakage-free, prospective validation will need to look like before such hybrid models reach the bedside. Westvik et al. took a related approach in very preterm infants, estimating serum phosphate levels from routinely collected blood gas, anthropometric, and nutritional data, with small-for-gestational-age status emerging as the dominant risk factor for early hypophosphatemia. Both studies point to the same opportunity that has long motivated model-informed dosing: extracting more clinical value from data already being collected, rather than adding burden to already vulnerable patients. Recent physiologically based pharmacokinetic modeling of pediatric antibiotic dosing offers a complementary, developmentally grounded route to the same goal (Tanaka et al., 2025).

## Digital technologies for maternal and perioperative monitoring

A second cluster of studies extends prediction into real-time obstetric care. Li et al. evaluated a consumer smartwatch for detecting spinal anesthesia-induced hypotension during cesarean delivery, finding moderate accuracy at a clinically relevant threshold (area under the receiver operating characteristic curve [AUC] 0.87, sensitivity 79.2%, specificity 82.0%) but reduced sensitivity at a milder one, appropriately positioning the device as a screening aid rather than a diagnostic replacement. Wang and He developed logistic regression, Naive Bayes, random forest, support vector machine, and XGBoost models to predict intrapartum labor analgesia efficacy from preoperative demographic, clinical, ultrasound, and psychological indicators in 137 parturients; their XGBoost model showed strong discrimination in training (AUC 0.875) that declined in validation (AUC 0.759), an instructive reminder that external testing must precede clinical use. He et al. studied nalbuphine-ropivacaine infiltration along the epidural puncture tract in a prospective cohort of 473 women undergoing cesarean delivery, reporting lower rates of post-neuraxial puncture-site pain than with ropivacaine infiltration alone, alongside an interpretable XGBoost risk model (AUC 0.912); as the authors themselves note, SHAP values describe statistical association within the model, not causal mechanism, so the treatment comparison still needs confirmation in an appropriately controlled trial.

## Building learning healthcare systems

None of the above is clinically useful without infrastructure that turns knowledge into action at the point of prescribing, drawing on the kind of routinely collected, real-world clinical data that increasingly underpins broader evaluations of drug safety and effectiveness (Corrigan-Curay et al., 2018). Wu et al. describe the construction of China’s first Pediatric Rational Medication Intelligent Decision System, a database of more than 400,000 entries across 1,030 medications covering dosing, interactions, infusion parameters, pharmacogenomics, and compatibility with traditional Chinese medicines. Embedded into the hospital information system with real-time access to patient biochemical and disease data, the system was associated with a significant reduction in higher-severity prescription alerts. This contribution is a reminder that predictive algorithms and curated, governed knowledge bases are not competing approaches but necessary complements; sophisticated models still depend on structured, pediatric-specific, continuously maintained medication data to reach the bedside. It also highlights the adoption, implementation, and governance of the underlying systems that support this application, with the intention of improving patient outcomes.

## Future directions and conclusion

These six contributions sit within a much broader shift already reshaping drug discovery and clinical trial design (Paul et al., 2021) and raising ethical and regulatory questions across the clinical pharmacology discipline (Petkovic et al., 2024); obstetric and pediatric applications carry that broader shift into two of medicine’s least evidence-rich populations. Across the six studies specifically, common priorities emerge: external validation across institutions, developmental stages, and healthcare systems; evaluation that extends beyond discrimination to calibration, transportability, workflow burden, and effect on patient outcomes; and transparent, explainable models with human clinical oversight retained wherever predictions influence dosing, monitoring, or intervention. These considerations carry strong emphasis when the populations affected are pregnant women, neonates, and children. The next challenge is translating these advances into routine pediatric and obstetric practice beyond specialist centers (Thompson et al., 2024).

The contributions to this Research Topic show that AI and digital health can strengthen obstetric and pediatric pharmacology. The next phase of this work should shift from retrospective prediction and single-center feasibility to prospective evaluation and interoperable implementation. Ultimately, the success of artificial intelligence in obstetric and pediatric pharmacology will not be measured solely by algorithmic performance, but by its ability to support safer, more effective, and more individualized pharmacotherapy for mothers and children. As confidence in these approaches increases, we can hopefully expect more routine deployment, with vigilance in place to ensure they continue to perform as expected as our knowledge and experience improve.
